# Probabilistic Neighborhood-Based Data Collection Algorithms for 3D Underwater Acoustic Sensor Networks

**DOI:** 10.3390/s17020316

**Published:** 2017-02-08

**Authors:** Guangjie Han, Shanshan Li, Chunsheng Zhu, Jinfang Jiang, Wenbo Zhang

**Affiliations:** 1 Department of Information and Communication Systems, Hohai University, 200 North Jinling Road, Changzhou 213022, China; lishanhhu@outlook.com (S.L.); jiangjinfang@hhu.edu.cn (J.J.); 2 Department of Electrical and Computer Engineering, The University of British Columbia, Vancouver, BC V6T 1Z4, Canada; cszhu@ece.ubc.ca; 3 School of Information Science and Engineering, Shenyang Ligong University, China; zhangwenbo@yeah.net

**Keywords:** Underwater Acoustic Sensor Networks, data collection, probabilistic neighborhood

## Abstract

Marine environmental monitoring provides crucial information and support for the exploitation, utilization, and protection of marine resources. With the rapid development of information technology, the development of three-dimensional underwater acoustic sensor networks (3D UASNs) provides a novel strategy to acquire marine environment information conveniently, efficiently and accurately. However, the specific propagation effects of acoustic communication channel lead to decreased successful information delivery probability with increased distance. Therefore, we investigate two probabilistic neighborhood-based data collection algorithms for 3D UASNs which are based on a probabilistic acoustic communication model instead of the traditional deterministic acoustic communication model. An autonomous underwater vehicle (AUV) is employed to traverse along the designed path to collect data from neighborhoods. For 3D UASNs without prior deployment knowledge, partitioning the network into grids can allow the AUV to visit the central location of each grid for data collection. For 3D UASNs in which the deployment knowledge is known in advance, the AUV only needs to visit several selected locations by constructing a minimum probabilistic neighborhood covering set to reduce data latency. Otherwise, by increasing the transmission rounds, our proposed algorithms can provide a tradeoff between data collection latency and information gain. These algorithms are compared with basic Nearest-neighbor Heuristic algorithm via simulations. Simulation analyses show that our proposed algorithms can efficiently reduce the average data collection completion time, corresponding to a decrease of data latency.

## 1. Introduction

As one of the most influential technological inventions in the field of information technology, theInternet of Things (IoT) has attracted worldwide attention [1,2]. Presently, IoT technology is widely used in various areas such as smart grid [3], smart home [4], military equipment logistics [5], intelligent medical treatment [6], ecological-environmental protection [7], and disaster warning [8]. The biggest breakthrough of IoT is providing a way to combine the physical world and information world to gradually change the interactions between humans and nature. IoT is regarded as the third wave of the global information industry after personal computer and Internet.

**Figure 1:**
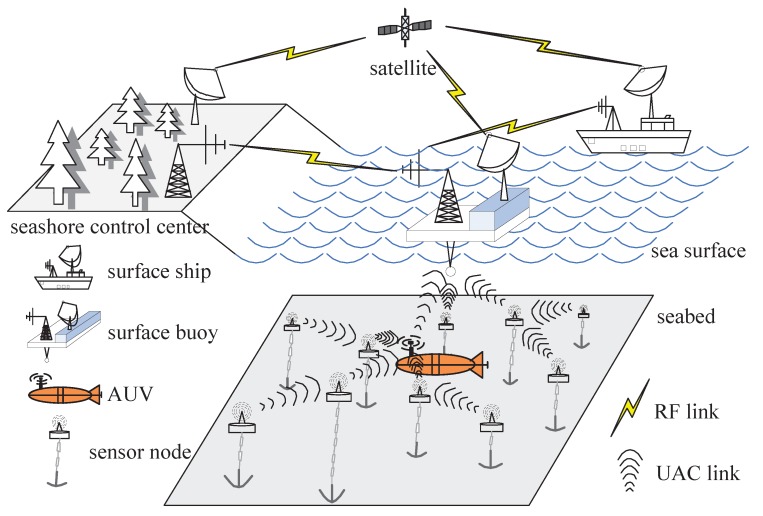
An application of 3D UASN for ocean monitoring.

One valuable application of IoT is utilizing three-dimension underwater acoustic sensor networks (3D UASNs) to perform marine environmental monitoring for the exploitation, utilization, and protection of marine resources [9]. Figure 1 shows a typical scenario of the 3D UASN application. Numerous sensor nodes are anchored at various depths in the ocean by buoys or cables, allowing information acquisition of the underwater surroundings. An autonomous underwater vehicle (AUV) is employed to assist information transmission. The monitoring information is finally delivered to the seashore control center via acoustic links and radio frequency (RF) links.

Data collection is a very typical application scenario of UASNs. With the advancement of secure underwater acoustic communication technology [10,11] and AUVs technology, AUVs have been widely adopted to facilitate data collection in UASNs. The utilization of AUVs can efficiently balance energy consumption of networks, reduce the energy costs of nodes, and thereby extend the survival time of UASNs. Furthermore, the use of AUVs can also address the frequent disconnection problem of networks caused by water currents. In the past few years, great advances have been made in the auxiliary data collection of AUVs for UASNs.

However, since most existing studies of underwater data collection are based on 2D scenarios, related schemes cannot be directly applied to 3D UASNs. In addition, because of the specific propagation effects resulting from the use of acoustic communication channels, the successful information delivery probability decreases progressively with distance. In this work, we investigate two probabilistic neighborhood-based AUV auxiliary data collection algorithms based on a probabilistic acoustic communication model for 3D UASNs.

The main contributions of this paper include: (1) design of the grid and probabilistic neighborhood-based data collection algorithm with layered-scan (GPN-LSCAN) for 3D UASNs with unknown deployment knowledge by partitioning the network into grids; (2) design of the probabilistic neighborhood covering set-based greedy heuristic algorithm (PNCS-GHA) for 3D UASNs with pre-known deployment knowledge; (3) extension of the probabilistic neighborhood-based minimum covering set construction algorithm to decrease data latency.

The remainder of this paper is organized as follows. Section 2 presents the related work regarding the underwater data collection problem. Section 3 describes the network model and probabilistic communication model in our paper. Section 4 investigates two probabilistic neighborhood-based data collection algorithms for 3D UASNs. Simulation results and performance evaluations are shown in Section 5. Section 6 presents our conclusions.

## 2. Related Work

There are many issues impeding the optimal deployment of UASNs for data acquisition in the field of marine environmental monitoring and surveillance. Due to a large amount of absorption and scattering losses, underwater applications mainly use acoustic communications instead of radio communications. Inevitably, the utilization of acoustic channels presents several challenges in data collection due to long propagation latency, narrow bandwidth, and a high bit error rate. Therefore, multi-hop transmission techniques from normal nodes to a pre-deployed sink have been preferred methods applied to UASN data collection [12,13].

Some sensor nodes in multi-hop networks are selected as relay nodes to forward packets from other nodes that are far from the sink. These relay nodes exhaust their energies more rapidly than the other nodes, which leads to extremely uneven energy consumption. In addition, the multi-hop communications of large-scale networks usually lead to excessive consumption of energy. Additionally, due to the limitation of acoustic communication in UASNs, it is challenging to ensure network connectivity, so traditional data collection methods based on multi-hop communication often fail.

To address the problem of link disconnection caused by water current, Kishigami et al. proposed a data collection scheme to repair the broken link by using controlled mobile nodes as relay nodes in [14]. If the distance between two neighboring nodes is larger than a threshold, the sink node will guide the mobile node to the central location of the corresponding link to recover the broken link. This scheme can efficiently address the disconnected network problem caused by the specific underwater environment, however, the issues of large scaled networks and uneven energy consumption were not addressed in this proposal.

Next, Anupama et al. in [15] investigated a geographical location-based clustering algorithm LCAD for data collection in 3D UASNs. According to the optimal horizontal and vertical transmission range derived experimentally, they divided the entire network into 3D grids that were 30 × 40 × 500 m^3^ in size. Each grid forms a cluster with a single cluster-head. The cluster-head nodes collect data from their cluster member nodes and then transfer the collected data to the sink by inter-cluster multi-hop transmission. This scheme can effectively reduce the energy consumption of normal nodes, but it does not address the overuse and the energy earlier exhausted problems of the cluster head nodes nearest the sink node.

Similarly, Domingo et al. in [16] presented an efficient clustering routing protocol in which data is transmitted to a destination node with a single hop. Though clustering is an effective method to optimize the total energy consumption in large-scale networks, the issue of uneven consumption of energy remains a problem for cluster-heads, which increases the need for a mobile element to collect data from neighborhoods in UASNs.

Kartha et al. in [17,18] designed a mobile sink-based model for data collection in UASNs and analyzed the network performance indicators of this model, including energy consumption, packet delivery ratio, network lifetime, and message delay. The results of analysis and simulation showed that the mobile sink auxiliary data collection for UASNs can effectively improve the data delivery ratio and optimize node energy usage to prolong the lifetime of UASNs.

In [19], Ilyas et al. proposed the AUV-aided routing protocol AEDG for reliable data collection in UASNs. They used a Shortest Path Tree algorithm to gather data of nodes to gateways, and then utilized an AUV to collect the data from the gateways. Though this model can effectively balance energy consumption and prolong lifetime of network, in their methods, the communication radius is presumed to be deterministic, although in practive, data transmission in UASN, is probabilistic and degrades with distance.

Recently, in [20] and [21], Hollinger et al. described a heuristic approximation algorithm for underwater data collection using an AUV as the mobile sink. They employed a probabilistic acoustic communication model which progressively decreases with distance. An AUV treated as the mobile sink was utilized to travel along the designed Traveling Salesman Problem-tour (TSP-tour) and stop at specific locations for data collection. Ultimately, a time-division multiple access (TDMA) protocol was employed, which allows the AUV to collect data packets after identifying nodes within current probabilistic neighborhood. This scheme can efficiently save transmission energy costs, reduce data delay, and improve data collection performance of UASNs. However, the authors did not examine resolution for 3D UASNs.

As summarized above, underwater data collection has been preliminarily studied, but most of the studies rely on ideal conditions or 2D UASNs. Therefore, there remains a significant need for additional research to address the mobile data collection problem for 3D UASNs.

## 3. Preliminaries

### 3.1. Network Model

It is assumed that numerous isomorphic nodes, which are constrained by restricted resources such as weakened battery, limited data storage space, and inefficient data manipulation, are randomly distributed in a 3D UASN to monitor the underwater surroundings. These nodes are considered static and they can obtain their locations by existing localization algorithms [22,23,24]. The nodes are equipped with acoustic communication modules to communicate with other devices, but the transmission range is limited. Additionally, a rechargeable AUV with autonomous navigation capability is employed as a mobile sink to collect the sensed data from nodes. A surface control center is deployed to execute centralized computing and recharge the AUV. The AUV starts from surface control center, travels along a designed path and moves around some specific locations named tour-points for data collection. The capabilities of bulk data communications of the sensor nodes are limited to single-hop transmission to the nearby AUV, to reduce energy consumption. As the AUV approaches a predefined location, it schedules a TDMA channel access protocol for data collection. At that point, the data of nodes within the collection range is transferred to the AUV and buffered there for further processing.

### 3.2. Communication Model

In this paper, we employ a probabilistic underwater acoustic communication model as described in [25,26]. This model takes account of the frequency and distance-dependent signal attenuation in water, as well as the noise disturbances in the underwater environment, such as turbulence, shipping activity, thermal noise, and wind.

Due to the absorption of the medium itself, the extension of wavefront during acoustic propagation, and scattering phenomenon caused by components such as magnesium sulfate and magnesium borate in the seawater, high speed communications over underwater acoustic channels are complicated but can be characterized by path losses. Based on a given distance *d* and frequency *f* (in kHz), the path loss can be calculated by an empirical formula given in [27]:

(1) $$A(d,f)=A_{0}d^{k}a{\left(f\right)}^{d}$$

where $A_{0}$ is a normalizing constant, and *k* is the spreading loss with typically used values of k=1 for cylindrical spreading, k=1.5 for practical spreading, and k=2 for spherical spreading. Additionally, a(f) represents the absorption coefficient (in dB re km), which can be described by the Thorp’s formula empirically as [28]:

(2) $$10\log a(f)=0.11\frac{f^{2}}{1+f^{2}}+44\frac{f^{2}}{4100+f^{2}}+2.75\times 10^{-4}f^{2}+0.003$$

The ambient noise is influenced by many factors: turbulence, thermal noise, shipping activity, and wind-driven waves. The noise can be finally modeled as [27]:

(3) $$10\log N_{t}\left(f\right)=17-30\log f$$

(4) $$10\log N_{th}\left(f\right)=-15+20\log f$$

(5) $$10\log N_{s}\left(f\right)=40+20\left(s-0.5\right)+26\log f-60\log\left(f+0.03\right)$$

(6) $$10\log N_{w}\left(f\right)=50+7.5w^{1/2}+20\log f-40\log\left(f+0.4\right)$$

where *s* ranging from 0 to 1 represents the surface shipping activity for low or high activity, and *w* is the speed of wind (in m re sec). The final noise level is the summation of these four factors:

(7) $$N(f)=N_{t}\left(f\right)+N_{s}\left(f\right)+N_{w}\left(f\right)+N_{th}\left(f\right)$$

Next, for moderate transmitted power *P* and signaling bandwidths *B*, the signal-to-noise ratio (SNR) at the receiver at a frequency *f* and distance *d* can be expressed as:

(8) $$SNR(d,f)=\frac{P/A(d,f)}{N(f)B}$$

Using the underwater acoustic communication model described above, a symbol error rate based on a SNR can be approximated as [25]:

(9) $$P_{e}=\frac{1}{4SNR}$$

For a packet with *N* symbols, the error probability of transmission is then:

(10) $$P_{packet}=1-{\left(1-P_{e}\right)}^{N}$$

Ultimately, for a packet with *N* symbols transmitted *m* rounds, the successful transmission probability can then be computed as:

(11) $$P=1-P_{packet}^{m}$$

Above all, the successful transmission probability is eventually a function of the transmission frequency, channel bandwidth, transmitted power, turbulence, shipping activity, thermal noise, wind speed, and transmission rounds.

## 4. Proposed Data Collection Algorithms

Here, we adopt the AUV auxiliary data collection method for 3D UASNs. By transferring the network energy consumption to the AUV, this method can effectively optimize the energy usage of the whole network, and thereby lengthen the network lifetime. However, due to the large scale of 3D UASNs and the limited velocity of the AUV, the mobile element auxiliary data collection often introduces long data delay.

In order to reduce data delay for mobile data collection in 3D UASNs, we propose two probabilistic neighborhood-based data collection algorithms: grid and probabilistic neighborhood-based data collection algorithm with layered-scan (GPN-LSCAN), and probabilistic neighborhood covering set-based greedy heuristic algorithm (PNCS-GHA). The GPN-LSCAN is appropriate for 3D UASNs without known sensor nodes’ locations. In contrast, PNCS-GHA is suitable for a 3D network in which the deployment information is known in advance. In our proposed algorithms, there is no need for the AUV to traverse all the nodes for data collection, allowing the efficient reduction of data collection completion time. Both algorithms are based on probabilistic neighborhoods defined as follows.

Based on the special communication model that has been discussed in Section 3, we define a *probabilistic neighborhood* Ψ_*i*_ which covers all spaces where the successful information delivery probability to node *i* is over *p*. The value of *p* ∈ [0,1] represents the conservative value of the successful information delivery to guarantee information gain. As *p* approaches 1, it will approach deterministic transmission. As *p* approaches 0, the AUV may need to access the node multiple times to collect the data. In the next subsections, we discuss the two probabilistic neighborhood-based data collection algorithms in detail.

### 4.1. Grid and Probabilistic Neighborhood-Based Data Collection Algorithm with Layered-Scan(GPN-LSCAN)

For 3D UASNs without prior deployment knowledge, we propose the GPN-LSCAN algorithm. This algorithm partitions the network into grids, which allows the AUV to visit the central location of each grid for data collection. Generally, our GPN-LSCAN contains three phases. In the first phase, the network is partitioned into several same size grids according to the parameter *p* and the characteristics of the underwater acoustic network. Next, a traveling trajectory with layered-scan is planned for the AUV to traverse all the grids. In the last phase, the AUV travels along the specified path and schedules a TDMA-based multiple access control protocol to collect data from the whole network. We will illustrate the three phases in detail in the following parts.

#### 4.1.1. Network Partition Phase

**Figure 2:**
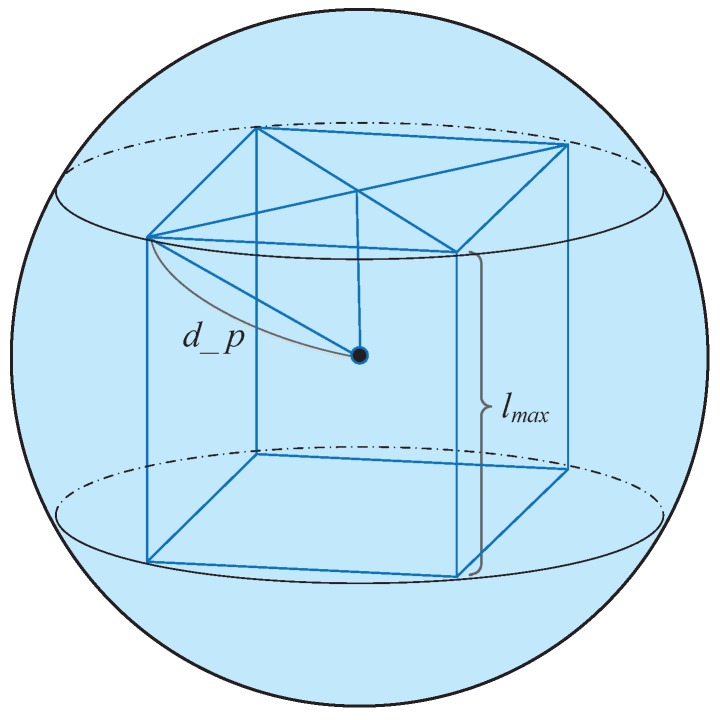
Grid size calculation in GPN-LSCAN.

**Figure 3:**
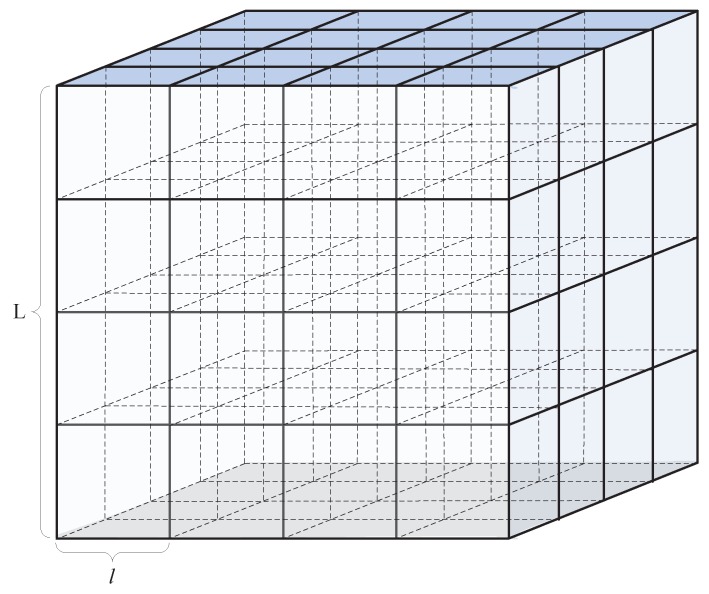
Network partition in GPN-LSCAN.

First, according to the equations discussed in Section 3, the control center computes the probabilistic neighborhood range d_p of the required parameter *p*. In order to ensure that all the nodes in one grid can send their storage data to the AUV, the grid must be fully included in the probabilistic neighborhood contour. As shown in Figure 2, the maximal unit cube is an inscribed regular hexahedron of the probabilistic neighborhood contour sphere. Clearly the final grid side length *l* can be calculated by:

(12) $$l_{max}=\frac{2\sqrt{3}d\_p}{3}$$

(13) $$k=\left\lceil \frac{L}{l_{max}}\right\rceil =\left\lceil \frac{\sqrt{3}L}{2d\_p}\right\rceil$$

(14) $$l=\frac{L}{k}=\frac{L}{\left\lceil \sqrt{3}L/2d\_p\right\rceil }$$

where $l_{max}$ is the side length of inscribed regular hexahedron, and *L* is the side length of network. Then, the 3D UASN is divided into k∗k∗k cubes with the side length of *l* as shown in Figure 3.

#### 4.1.2. AUV Trajectory Planning Phase

**Figure 4:**
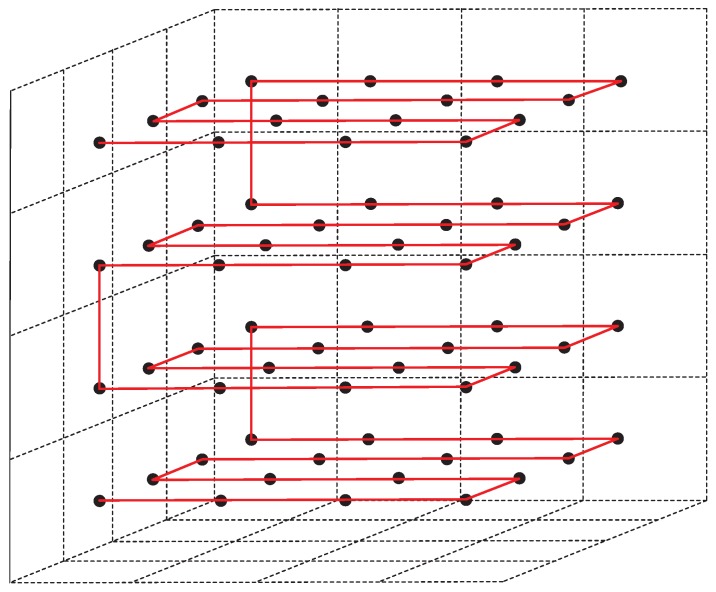
Layered-scan of the AUV.

After dividing the entire 3D network into several grids, the AUV executes a traveling path to collect data from the whole network. Five kinds of paths have been described, called Layered-Curve, Triple-Curve, Layered-Scan, Triple-Scan, and 3D-Hilbert that can fill a given cubic area. In [29], Cui et al. compared these five paths with difference parameters. The simulation results showed that, in these five kinds of proposed paths, Layered-Scan and 3D Hilbert showed the best performance in term of path length. Because here we only need to consider path length, we selected the Layered-Scan as the trajectory of the AUV in this paper. Dividing the 3D region into several layers along one axis, each layer is regarded as a 2D plane, which results in Layered-Scan paths as shown in Figure 4.

#### 4.1.3. Data Collection Phase

**Figure 5:**
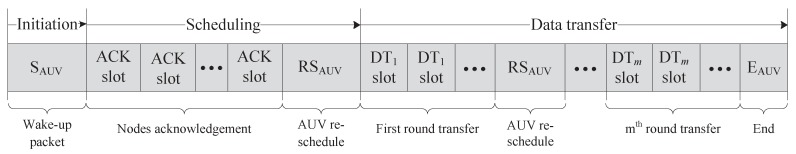
TDMA-based multiple access control protocol.

In this phase, the AUV travels along the specified path and moves around the specified locations to collect data from nodes within each probabilistic neighborhood. The residence time varies with the number of nodes within each probabilistic neighborhood and the value of transmission rounds adaptively. Then, a TDMA-based multiple access control protocol as shown in Figure 5 is utilized. The protocol mainly contains three phase illustrated as follow:

(1) *Initiation:* All the functional nodes which are randomly distributed in the water start in an inactive state. When the AUV approaches a predefined location, it broadcasts a wake-up control packet including initial schedules for the nodes within the probabilistic neighborhood. The high power wake-up control packet can trigger the nodes to enter an active state.

(2) *Scheduling:* Using the initial schedules, the nodes that received the wake-up packet send a small acknowledgement packet in response to the AUV. Then, the AUV transmits scheduling information for the next round of transmission to these nodes.

(3) *Data Transfer:* Using the updated scheduling information, the functional nodes within the current collect area transfer their data packets to the AUV. Once all transmissions in one round have occurred, the AUV re-schedules the nodes for the next round of data transmission. The total round of transmission is predefined. Finally, the AUV broadcasts a short packet to announce the end of data collection in current probabilistic neighborhood. Then the nodes reenter the inactive state.

### 4.2. Probabilistic Neighborhood Covering Set-Based Greedy Heuristic Algorithm (PNCS-GHA)

For 3D UASNs with pre-known deployment knowledge, we propose the PNCS-GHA for data collection. Similarly, our PNCS-GHA also contains three phases. In the first phase, we construct a minimum probabilistic neighborhood covering set as resident points according to the probabilistic neighborhood contour. Then, we employ the Nearest-neighbor Heuristic strategy which greedily moves to the nearest neighborhood to traverse all the covering set nodes to collect data within their probabilistic neighborhoods. After planning the data collection tour, the AUV travels along the specified path to collect data from the whole network. The AUV stores the visit status information of PNCS nodes. Once all the PNCS nodes have been visited, the AUV terminates current data collection travel and directly returns to the surface control center to prepare for the next travel. The third phase is highly similar to the data collection phase of the GPN-LSCAN, so we only discuss the probabilistic neighborhood covering set construction phase and AUV trajectory planning phase in detail.

#### 4.2.1. Probabilistic Neighborhood Covering Set Construction Phase

In our PNCS-GHA, all the locations of nodes are assumed to be pre-known by the control center. On the basis of the location information and the given *p*, a minimum probabilistic neighborhood covering set (PNCS) can be constructed by control center to shorten the path length of data collection tour. Algorithm 1 presents the main procedure of PNCS construction.
**Algorithm 1** Probabilistic neighborhood covering set construction**Input:** The candidate nodes set N=[1,2,…,n]; the locations of nodes in *N*; the parameter *p*;**Output:** The minimum probabilistic neighborhood covering set, *S*;
1:calculate the probabilistic neighborhood contour d_p according to the given *p* and equations in Section 3;2:**loop**3:  each node in *N* calculates its neighbor nodes set Nei(i) (also belong to *N*) within contour d_p, and its neighbor weight num(i), respectively;4:  $s_{j}$ = min {arg max {num(i)|i∈N}};5:  add $s_{j}$ into *S*;6:  remove $s_{j}$ and $Nei(s_{j})$ from candidate set *N*;7:  **if** N≠Φ **then**8:    repeat loop;9:  **end if**10:**end loop**


**Figure 6:**
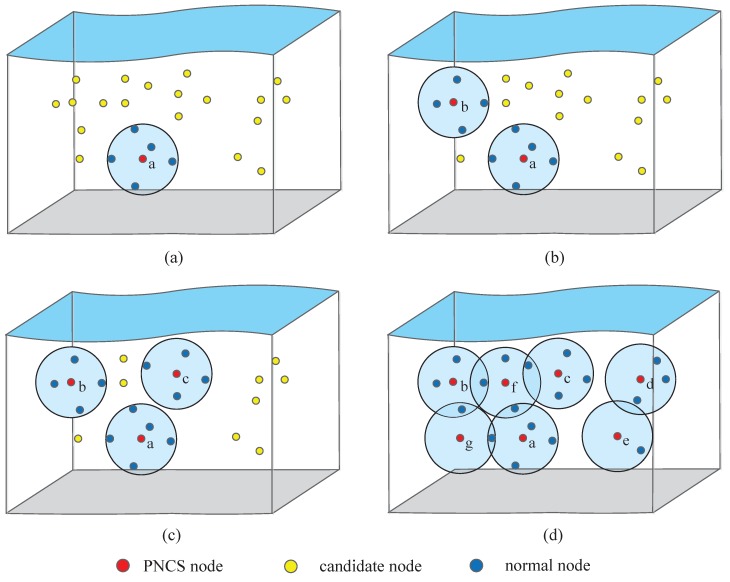
An example of PNCS construction (**a**) The first PNCS node selection; (**b**) The second PNCS node selection; (**c**) The third PNCS node selection; (**d**) The final PNCS construction.

We illustrate an example for our minimum covering set construction mechanism in Figure 6. First, every node in the 3D network is joined into the candidate set *N*. Each candidate node calculates its neighbor node set and neighbor weight. Then the node with the maximum neighbor weight will be inserted into the covering set. As shown in Figure 6a, the node *a* has the biggest neighbor weight, so node *a* is first added into *S*, i.e., S={a}. Then the node *a* and its neighbor nodes set are removed from the candidate set *N*. As the candidate set *N* is not empty, the nodes in current *N* recalculate their neighbor nodes sets and neighbors weights. Then as shown in Figure 6b, the node *b* is added into *S*, i.e., S={a,b}. The loop will be repeated until N=ϕ, the final probabilistic neighborhood covering set *S* as shown in Figure 6d is S={a,b,c,d,e,f,g}.

#### 4.2.2. AUV Trajectory Planning Phase

Once the PNCS has been constructed, we need to determine a path for the AUV to traverse all the PNCS nodes. This is a typical NP-hard problem and is quite similar to TSP. At present, some classic heuristic strategies have been proposed to solve TSP, such as Nearest-neighbor Heuristic, Heuristics of Christofides, Minimum Spanning Tree, and Random Insertion [30]. Here, in order to reduce the excessive complexity of 3D path planning and shorten the path length, we employ the Nearest-neighbor Heuristic, which is easily and efficiently extended to 3D space, for the AUV to traverse every node in the constructed PNCS. Algorithm 2 presents the main procedure of the PNCS-based Nearest-neighbor Heuristic trajectory planning algorithm. Firstly, the visit status of each PNCS node is set to be false, represents that this node hasn’t been visited by the AUV. Next, among those PNCS nodes whose visit statuses are false, the nearest node of the AUV is greedily selected as the next stay point for data collection, and the visit status of this node is set to be true. Then, the greedy heuristic procedure is repeated until all the PNCS nodes have been visited.
**Algorithm 2** Planning the AUV trajectory in PNCS-GHA**Input:** The constructed probabilistic neighborhood covering set *S*; the locations of nodes in *S*;**Output:** The traveling path of the AUV, *P*;
1:*m* = |S|;2:**for** *i* = 1 to *m* **do**3:    visit(i) = false;4:    $d_{0}\left(i\right)$ = distance from S(i) to the surface control center;5:**end for**6:curr = {arg min {$d_{0}\left(i\right)|i=1,2,\ldots ,m$}};7:visit(curr) = true;8:visitNum = 1;9:*P* = {S(curr)};10:**while** visitNum<m **do**11:    **for** *i* = 1 to *m* **do**12:        **if** visit(i) = false **then**13:           $d_{curr}\left(i\right)$ = distance from S(i) to S(curr);14:        **else**15:           $d_{curr}\left(i\right)$ = ∞;16:        **end if**17:    **end for**18:    curr = {arg min {$d_{curr}\left(i\right)|i=1,2,\ldots ,m$}};19:    visit(curr) = true;20:    visitNum = visitNum +1;21:    *P* = {*P*; S(curr)};22:**end while**


## 5. Performance Evaluation

To estimate the performance of our proposed algorithms, a 3D UASN with random deployments of *n* nodes over an area of size 1000 × 1000 × 1000 m^3^ was simulated in MATLAB. Table 1 gives the network parameters employed in our simulation model in detail. In order to observe the performance more intuitively, we select three performance parameters: the information gain *G*, the data collection completion time *T* (corresponding to data delay), and the gain to cost ratio *η*. These parameters can be calculated by Equations (15)–(17):

(15) $$G=\sum_{i=1}^{N}\left[Q*{\left(1-P_{packet}\left(i\right)\right)}^{m}\right)]$$

(16) $$T=L_{path}/v_{AUV}+\sum_{j=1}^{M}\left[T_{residence}\left(j\right)\right]$$

(17) η=G/T

where *Q* is the packet size of each node, $P_{packet}\left(i\right)$ is the probability of transmission error of node *i* (see Section 3), *m* is the transmission round, the $L_{path}$ is the total length of traversal path, *M* is the number of stay points, and $T_{residence}\left(j\right)$ is the residence time of the AUV in each stay point *j*. The residence time is the sum of initiation time, scheduling time, transfer time, and additional compensatory time. It varies with the number of nodes within each probabilistic neighborhood and the value of transmission rounds adaptively. As we can see from the formulations, the gain to cost ratio *η* is to evaluate data collection performance as a tradeoff between the data collection completion time and information gain. The greater the value of *η* is, the better the data collection performance.

### 5.1. Data Collection Performance Comparison

In previous work, we proposed two probabilistic neighborhood-based data collection algorithms for 3D UASNs. To evaluate the performance of our algorithms, we compared them with one of the most widely used 3D path planning method, the basic Nearest-neighbor Heuristic. In the basic Nearest-neighbor Heuristic, the AUV moves to the nearest node without any probabilistic neighborhoods, which means that the AUV needs to visit all nodes. Simulations were run for the three schemes varying parameters *p* and *n*. The parameter *p* represents the design decision when implementing the probabilistic neighborhood contour based on our proposed algorithms. The value of parameter *p* indicates the size of probabilistic neighborhoods, and the higher the parameter *p* is, the smaller the size of the probabilistic neighborhoods. The moving speed of the AUV is set to 5 m/s.

**Figure 7:**
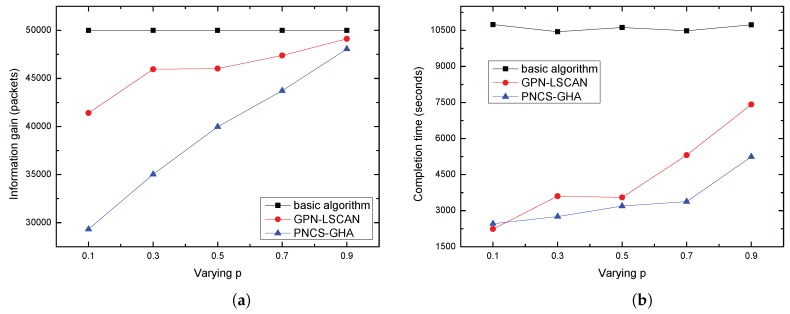
(**a**) Average information gain VS *p*; (**b**) Average completion time VS *p*.

(1) *The effects of p*: In our simulations, a value of *p* = 0.9 corresponds to approximately 150 m of neighborhood size, *p* = 0.5 to approximately 240 m of neighborhood size, and *p* = 0.1 to approximately 300 m of neighborhood size, respectively. The number of nodes was set to be 500. Figure 7 shows the average information gain and the average mission time with variable parameter *p*. As expected, with the increase of parameter *p*, corresponding to increased successful data transmission probability, there was increased information gain, as shown in Figure 6a. As data collection field at a tour point of GPN-LSCAN is a inscribed regular hexahedron and the volume accounts for only about 3/8 of the probabilistic neighborhood contour sphere, the information gain of GPN-LSCAN is higher than that of PNCS-GHA. In addition, as shown in Figure 6b, the average data collection completion time increases with the increase of *p*. This is because as the value of *p* increases, the size of neighborhoods decreased, so the path length of the AUV increased, requiring additional time for data collection completion.

**Figure 8:**
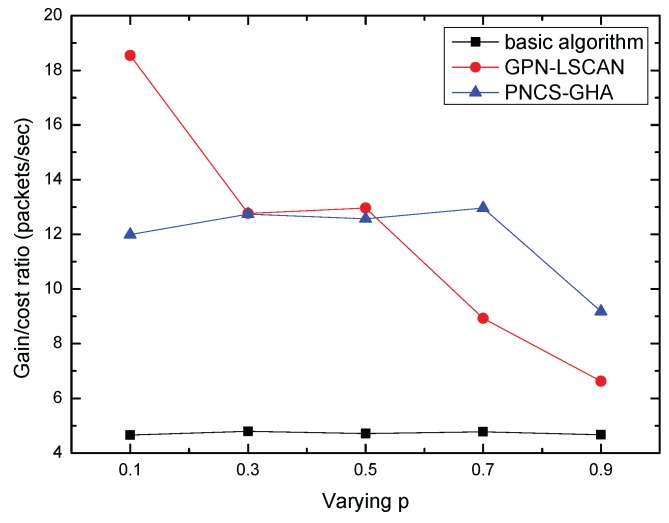
Average gain to cost ratio VS *p*.

Figure 8 shows the gain to cost ratio of our proposed algorithms. Both our GPN-LSCAN and PNCS-GHA perform much better than the basic algorithm. We also note that with a lower required probability, GPN-LSCAN showed a higher gain to cost ratio than PNCS-GHA, but at a high *p*, the gain to cost ratio of GPN-LSCAN was lower than that of PNCS-GHA. This is because when the neighborhood size is large enough, the path length of GPN-LSCAN approaches that of the PNCS-GHA. When the grid size is small, the path length is significantly longer.

**Figure 9:**
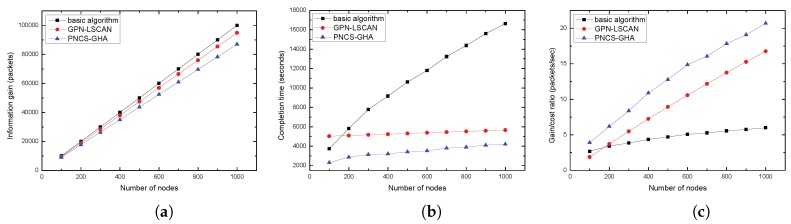
(**a**) Average information gain VS *n*; (**b**) Average completion time VS *n*; (**c**) Average gain to cost ratio VS *n*.

(2) *The effects of the number of nodes n*: In this part, the parameter *p* was set to be 0.7. Figure 9a shows the variation of the average information gain for different *n* arguments in the GPN-LSCAN, PNCS-GHA, and basic algorithm. In both the GPN-LSCAN and PNCS-GHA models, the average information gain significantly increased as *n* increased. Figure 9b validates the advantages of our proposed algorithm in average data collection completion time. Since the AUVs in GPN-LSCAN and PNCS-GHA do not need to visit every node in the 3D UASN, the average data collection completion time in our proposed algorithms are obviously shorter than that of the basic greedy algorithm. Additionally, we also find that when *n* increased, the average data collection completion time in our proposed algorithms increased more slowly than what occurs when using the basic algorithm without neighborhood. This trend is interpreted below. Obviously, increases in *n* typically cause longer paths for the AUV. However, as the path length of the GPN-LSCAN model is influenced by the size of network and neighborhood, and the path length of the PNCS-GHA is influenced by a combined impact of neighborhood size and nodes number, the increased number of nodes has less impact on the path costs of our proposed algorithms than on the basic algorithm without any neighborhoods. Accordingly, the costs of our two algorithms become much lower than the cost of the basic algorithm as *n* increases. In Figure 9c, we note that, when the *n* increases, the gain to cost ratio in our proposed algorithms becomes enormously higher than that of the basic algorithm without any probabilistic neighborhoods.

### 5.2. Performance Comparison for PNCS-GHA under Different Transmission Rounds

In this subsection, the *n* was set to 100 and the AUV’s moving velocity was set to 2.5 m/s. The value of transmission rounds was changed for different values of parameter *p* to determine how the PNCS-GHA performs. A minimum probabilistic neighborhood covering set was constructed through simulation, and then the PNCS-based data collection path for the AUV was generated to traverse all the neighborhoods in the constructed covering set. We do not consider the variable *n* here, but concentrate on the impact of transmission rounds. Simulations were run for the PNCS-GHA of different neighborhood sizes with variable transmission rounds.

**Figure 10:**
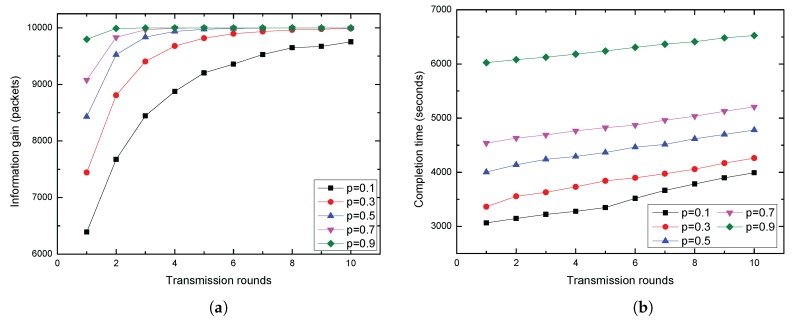
(**a**) Average information gain VS transmission rounds; (**b**) Average completion time VS transmission rounds.

Figure 10 shows the average information gain and the average data collection completion time for variable parameter *p* with different transmission rounds. With increased transmission rounds, the average information gain as shown in Figure 10a gradually approaches saturation and the average data collection completion time in Figure 10b increased steadily. In addition, the information gain and data collection completion time increased with the increase of *p*.

**Figure 11:**
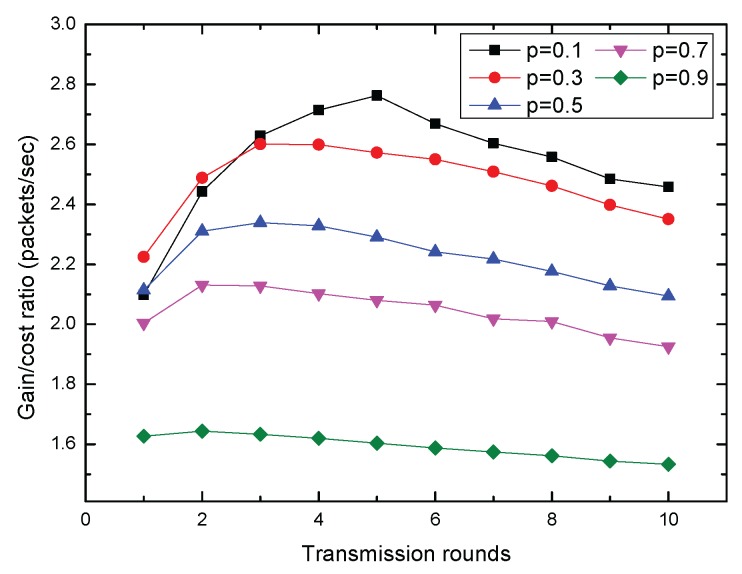
Tradeoff between completion time and information gain.

In Figure 11, we show the gain to cost ratio for different *p* values with variable transmission rounds. It can be seen that with increased transmission rounds, the gain to cost ratio decreased after increasing initially. For different values of parameter *p*, the gain to cost ratio reached the maximum on individual transmission rounds. When the *transmissionrounds* = 5 and *p* = 0.1, the gain to cost ratio reached a global maximum. It is important to point out that the gain to cost ratio reached the maximum on distinct transmission rounds due to the communication parameter setting.

**Figure 12:**
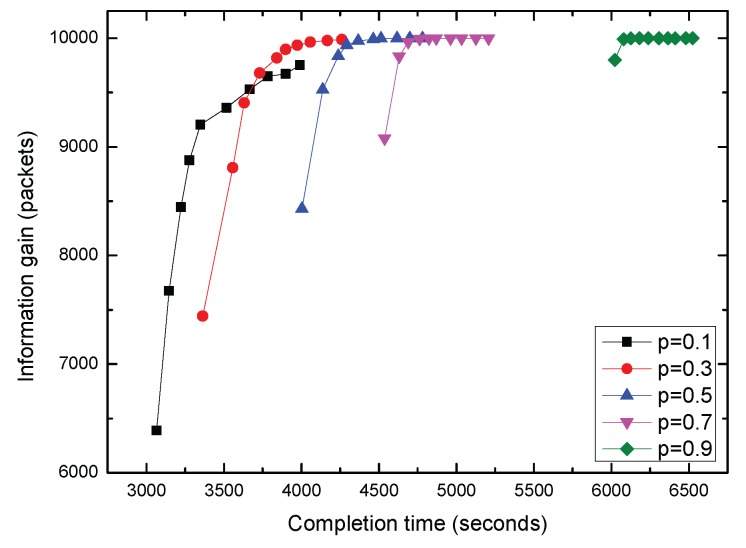
Tradeoff between completion time and information gain.

Finally, in Figure 12, we present an intuitive chart of information gain versus data collection completion time for variable parameter *p* and transmission rounds. By increasing the transmission rounds, our proposed algorithms allow a tradeoff between data collection completion time and information gain.

## 6. Conclusions

Currently, 3D UASN technology is widely applied in marine environment monitoring to explore marine resources. In this paper, two probabilistic neighborhood-based AUV auxiliary data collection algorithms were investigated for 3D UASNs, aiming to reduce data delay and prolong network longevity. Our results indicate that the GPN-LSCAN is appropriate for UASNs in which the locations of sensor nodes are previously unknown by AUV. In contrast, PNCS-GHA is more suitable for a network in which deployment information is known in advance. In our proposed algorithms, there is no need for AUV to traverse all the nodes for data collection, but instead, the sampling of a subset of sites can be performed to efficiently shorten the data collection completion time. By increasing the transmission rounds, our proposed algorithms provide a tradeoff between the data collection completion time and information gain. These schemes are compared with a basic strategy via simulations. Simulation analyses demonstrate that our proposed algorithms exhibit superior performance for gain to cost ratio and average data collection completion time, corresponding to a decrease in data latency.

## Figures and Tables

**Table 1:** Parameter Configuration.

Parameters	Value
Network Size	1000 × 1000 × 1000 m^3^
Number of Nodes	100–1000
Parameter *p*	0.9, 0.7, 0.5, 0.3, 0.1
Probabilistic Neighborhood Contour	150, 210, 240, 270, 300 (m)
Moving Speed of AUV	2.5, 5 (m/s)
Transmission Frequency	10 kHz
Transmission Power	1 W
Transmission Round	1–10
Bandwidth	4 kHz
Number of Each Node’s Data Packets	100 packets

## References

[1] Zhu C., Leung V.C.M., Shu L., Ngai E.C.H. (2015). Green Internet of Things for Smart World. IEEE Access.

[2] Qiu T., Chen N., Li K., Qiao D., Fu Z. (2017). Heterogeneous Ad Hoc Networks: Architectures, Advances and Challenges. Ad Hoc Networks.

[3] Dong C., Zhou J., Wang M. (2015). Application of the Internet of Things Technology in Smart Grid. Adv. Social Sci. Educ. Hum. Res..

[4] Chang C., Kuo C., Chen J., Wang T. (2015). Design and Implementation of an IoT Access Point for Smart Home. Appl. Sci..

[5] Liang F., Bai H., Liu G. (2014). Application of Internet of Things in Military Equipment Logistics. Appl. Mech. Mater..

[6] Ma Y., Zhang Y., Dung O.M. (2015). Health Internet of Things: Recent Applications and Outlook. J. Internet Technol..

[7] Wu D., Dai L., Dai H. (2015). Application of Internet of Thing Technology in Ecological-Environmental Protection of Long-Distance Water Diversion Project. Appl. Mech. Mater..

[8] Spalazzi L., Taccari G., Bernardini A. An Internet of Things Ontology for Earthquake Emergency Evaluation and Response. Proceedings of the International Conference on Collaboration Technologies and Systems.

[9] Zhou Z., Yao B., Xing R., Shu L., Bu S. (2016). E-CARP: An Energy Efficient Routing Protocol for UWSNs in the Internet of Underwater Things. IEEE Sens. J..

[10] Han G., Jiang J., Sun N., Shu L. (2015). Secure Communication for Underwater Acoustic Sensor Networks. IEEE Commun. Mag..

[11] Han G., Jiang J., Shu L., Guizani M. (2015). An Attack-Resistant Trust Model based on Multidimensional trust Metrics in Underwater Acoustic Sensor Networks. IEEE Trans. Mob. Comput..

[12] Ayaz M., Baig I., Abdullah A., Faya I. (2011). A Survery on Routing Techniques in Underwater Wireless Sensor Networks. Int. J. Comput. Appl..

[13] Han G., Jiang J., Bao N., Wan L., Guizani M. (2015). Routing Protocols for Underwater Wireless Sensor Networks. IEEE Commun. Mag..

[14] Kishigami W., Tanigawa Y., Tode H. Robust Data Gathering Method Using Controlled Mobility in Underwater Sensor Network. Proceedings of the IEEE International Conference on Sensing, Communication, and Networking (SECON).

[15] Anupama K.R., Sasidharan A., Vadlamani S. A Location-Based Clustering Algorithm for Data Gathering in 3D Underwater Wireless Sensor Networks. Proceedings of the International Symposium on Telecommunications.

[16] Domingo M.C. (2011). A Distributed Energy-Aware Routing Protocol for Underwater Wireless Sensor Networks. Wirel. Pers. Commun..

[17] Kartha J.J., Jabbar A., Baburaj A., Jacob L. Maximum Lifetime Routing in Underwater Sensor Networks using Mobile Sink for Delay-Tolerant Applications. Proceedings of the TENCON IEEE Region 10 Conference Proceedings.

[18] Kartha J.J., Jacob L. (2015). Delay and Lifetime Performance of Underwater Wireless Sensor Networks with Mobile Element Based Data Collection. Int. J. Distrib. Sens. Netw..

[19] Ilyas N., Alghamdi T.A., Farooq M.N. (2015). AEDG: AUV-aided Efficient Data Gathering Routing Protocol for Underwater Wireless Sensor Networks. Procedia Comput. Sci..

[20] Hollinger G.A., Choudhary S., Qarabaqi P., Murphy C., Mithra U., Sukhatme G.S., Stojanovic M., Singh H., Hover F. (2012). Underwater Data Collection using Robotic Sensor Networks. IEEE J. Sel. Areas Commun..

[21] Hollinger G.A., Mitra U., Sukhatme G. Autonomous Data Collection from Underwater Sensor Networks using Acoustic Communication. Proceedings of the IEEE International Conference on Intelligent Robots and Systems.

[22] Han G., Liu L., Jiang J., Shu L., Rodrigues J.J.P.C. (2016). A Collaborative Secure Localization Algorithm Based on Trust Model in Underwater Wireless Sensor Networks. Sensors.

[23] Han G., Jiang J., Zhang C., Duong T.Q., Guizani M., Karagiannidis G. (2016). A Survey on Mobile Anchors Assisted Localization in Wireless Sensor Networks. IEEE Commun. Surv. Tutorials.

[24] Han G., Zhang C., Shu L., Rodrigues J.J.P.C. (2015). Impacts of Deployment Strategies on Localization Performances in Underwater Acoustic Sensor Networks. IEEE Trans. Ind. Electron..

[25] Hollinger G.A., Yerramalli S., Singh S., Mitra U., Sukhatme G.S. Distributed Coordination and Data Fusion for Underwater Search. Proceedings of the IEEE International Conference on Robotics and Automation.

[26] Arrichiello F., Liu D.N., Yerramalli S., Pereira A., Das J., Mitra U., Sukhatme G.S. Effects of Underwater Communication Constraints on the Control of Marine Robot Teams. Proceedings of the International Conference on Robot Communication and Coordination.

[27] Stojanovic M. On the Relationship between Capacity and Distance in an Underwater Acoustic Communication Channel. Proceedings of the First ACM International Workshop on Underwater Networks.

[28] Berkhovskikh L., Lysanov Y. (1982). Fundamentals of Ocean Acoustics.

[29] Cui H., Wang Y., Lv J. (2012). Path Planning of Mobile Anchor in Three-dimensional Wireless Sensor Networks for Localization. J. Inf. Comput. Sci..

[30] Gong W., Li M. (2014). Comparison of Heuristics for Resolving the Traveling Salesman Problem with Information Technology. Adv. Mater. Res..

